# MicroRNA-211/BDNF axis regulates LPS-induced proliferation of normal human astrocyte through PI3K/AKT pathway

**DOI:** 10.1042/BSR20170755

**Published:** 2017-08-21

**Authors:** Kexiang Zhang, Song Wu, Zhiyue Li, Jiahui Zhou

**Affiliations:** Department of Orthopedics, The Third Xiangya Hospital, Central South University, Changsha 410013, China

**Keywords:** Brain-derived Neurotrophic Factor (BDNF), miR-211, normal human astrocyte (NHA), proliferation, spinal cord injury (SCI)

## Abstract

Spinal cord injury (SCI) makes a major contribution to disability and deaths worldwide. Reactive astrogliosis, a typical feature after SCI, which undergoes varying molecular and morphological changes, is ubiquitous but poorly understood. Reactive astrogliosis contributes to glial scar formation that impedes axonal regeneration. Brain-derived neurotrophic factor (BDNF), a well-established neurotrophic factor, exerts neuroprotective and growth-promoting effects on a variety of neuronal populations after injury. In the present study, by using LPS-induced *in vitro* injury model of astroglial cultures, we observed a high expression of interleukin (IL)-6, IL-1β, and BDNF in LPS-stimulated normal human astrocytes (NHAs). BDNF significantly promoted NHA proliferation. Further, online tools were employed to screen the candidate miRNAs which might directly target BDNF to inhibit its expression. Amongst the candidate miRNAs, *miR-211* expression was down-regulated by LPS stimulation in a dose-dependent manner. Through direct targetting, *miR-211* inhibited BDNF expression. Ectopic *miR-211* expression significantly suppressed NHA proliferation, as well as LPS-induced activation of PI3K/Akt pathway. In contrast, inhibition of *miR-211* expression significantly promoted NHA proliferation and LPS-induced activation of PI3K/Akt pathway. Taken together, *miR-211*/BDNF axis regulates LPS-induced NHA proliferation through PI3K/AKT pathway; *miR-211*/BDNF might serve as a promising target in the strategy against reactive astrocyte proliferation after SCI.

## Introduction

Spinal cord injury (SCI) makes a major contribution to disability and deaths worldwide with an annual incidence of 15–40 cases per million of the population and limited treatment options [1–3]. The loss of sensation and paralysis caused by SCI is irreversible, partially because injured axons encounter a series of inhibitory microenvironments which are non-permissive for growth [4–6].

Reactive astrogliosis, a typical feature after SCI, which undergoes varying molecular and morphological changes [7,8], is ubiquitous but poorly understood. Reactive astrogliosis can exert both beneficial and detrimental effects in a context-dependent manner determined by specific molecular signaling cascades [7,9]. Astrogliosis is a defense response of the central nervous system (CNS) to minimize primary damage and repair injured tissues, but it ultimately generates harmful effects by forming potent barriers to axon regeneration [10]. Reactive astrocytes participate in the process of glial scar formation [11] which could further inhibit axonal regeneration [12] and block nerve injury repair. Therefore, investigation of the mechanisms of reactive astrogliosis is beneficial to develop strategies for protection against SCI.

Brain-derived neurotrophic factor (BDNF) exerts neuroprotective and growth-promoting effects on a variety of neuronal populations after injury. Neuroprotective outcomes in particular may be attributed to downstream effects of BDNF and its receptor, tropomyosin-related kinase B (TrkB) signaling. Pro-apoptotic molecules such as glycogen synthase kinase 3 (GSK-3) [13,14], Bcl-2 associated death promoter (Bad) [15], and JNK [16] are inhibited by TrkB signaling via the PI3K/Akt pathway [13,17], allowing cells marked for death to survive. In addition, BDNF diminishes glutamate-induced apoptotic cell death [18]. In the present study, we evaluated the effects of BDFN in the regulation of NHA proliferation, and further investigated the underlying mechanism.

MiRNAs are endogenously derived, short (usually 22 nts in length), non-coding RNAs that regulate gene expression post-transcriptionally [19]. MiRNAs bind to the 3′-UTR of target mRNAs via imperfect base-pairing followed by either inhibition of translation or target degradation [20,21]. According to previous studies, miRNAs affect astrogliosis and astrocyte proliferation through targetting diverse downstream genes. Exogenous addition of anti-*miR-125b* to IL-6-stressed normal human astrocytes (NHAs) cultures attenuated glial cell proliferation and increased the expression of the cyclin-dependent kinase inhibitor 2A (CDKN2A), a target of *miR-125b* and a negative regulator of cell growth [22]. *MiR-146a* participates in the regulation of astroglial cell proliferation, the innate immune and inflammatory response [22–26]. These suggested that miRNAs may act as potent therapeutic targets in astrogliosis after SCI, most possibly through targetting their downstream genes.

In the present study, we have evaluated the expression and function of BDNF in LPS-stimulated NHAs. To further investigate the molecular mechanism, we employed online tools to search for the candidate miRNAs which might bind to the 3′-UTR of BDNF to inhibit its expression, and then assessed the likelihood of this miRNA–BDNF axis being a new therapeutic target. Taken together, we provided a novel experimental and theoretical basis of improving glial scar formation from the aspect of inhibiting NHA proliferation.

## Materials and methods

### Human astrocytes culture and transfection

Human astrocytes (NHAs, isolated from spinal cord) were obtained from the ScienCell (Cat# 1820, U.S.A.). Cells were cultured in astrocyte medium (Cat# 1801, ScienCell) supplemented with 15% FBS (GBICO, U.S.A.), 100 units/ml penicillin and 100 μg/ml streptomycin in a humidified incubator with 5% CO_2_ at 37°C. LPS (500 ng/ml, Sigma) was incubated with NHA cultures for 2 days before harvest.

*MiR-211* mimics or *miR-211* inhibitors (50 nmol/l, GenePharma, China) were transfected into the indicated target cells to achieve *miR-211* overexpression or *miR-211* inhibition by using Lipofectamine 2000 (Invitrogen). NC mimics and NC inhibitor (GenePharma, China) were used correspondingly as negative control. Si-BDNF (100 pmol/L) or pcDNA3.1/BDNF (1 μg/ml) was used to achieve knockdown of BDNF or BDNF overexpression (GeneCopoecia, China), si-NC and pcDNA3.1 were used correspondingly as negative control (GeneCopoecia, China).

### qRT-PCR

TRIzol reagent (Invitrogen) was used for total RNA extraction following the manufacturer’s instructions. Total RNA was reverse transcribed and the miScript Reverse Transcription kit (Qiagen, Germany) was used for *miR-211* qRT-PCR. qRT-PCR was performed on triplicate samples using miScript SYBR Green PCR Kit (Qiagen, Germany) on the ABI 7900HT Real-time PCR System (Applied Biosysterm, U.S.A.). The 2^−ΔΔ*C*^_t_ method was used to evaluate the relative expression and normalized to U6 expression. Data shown are representative of at least three independent experiments.

### Western blotting

RIPA buffer (Cell Signaling Tech., U.S.A.) was used to homogenize the cells. The protein levels of BDNF, PI3K, *p*-PI3K (Tyr^458^), Akt, and *p*-Akt (Ser^473^) in NHAs were detected by immunoblotting. Cells were harvested using scraper and lysed in 1% PMSF supplemented RIPA buffer. Cell lysates were centrifuged at 14000×***g*** for 15 min at 4°C and supernatants were subjected to Western blotting analysis. Protein was loaded on to SDS/PAGE minigel, and then transferred on to PVDF membrane. The blots were probed with the following antibodies: BDNF (Cat# EPR1292, Abcam, U.S.A.), GFAP (ab7260, Abcam), PI3K (Cat# M253, Abcam), *p*-PI3K (Tyr^458^, Cat# orb106105, Biorbyt Ltd., U.K.), Akt (Cat# Y89, Abcam), and *p*-Akt (Cat# EP2109Y, Abcam) at 4°C overnight, and incubated with HRP–conjugated secondary antibody (1:5000). Signals were visualized using ECL substrates (Millipore, U.S.A.). The protein expression was normalized to endogenous GAPDH.

### MTT cell viability assay

Cell viability was evaluated using a modified MTT assay. The viability of NHAs transfected with the indicated vectors or treated with TrkB-IgG (1 μg/ml) was assessed at four time points (at 0, 24, 48, and 72 h) after seeding 2 × 10^3^ transfected cells/well into 96-well culture plates. Briefly, quantitation of mitochondrial dehydrogenase activity was achieved via the enzymatic conversion of MTT (Sigma–Aldrich, MO, U.S.A.) to a colored formazan product. MTT (10 μl of 10 mg/ml) was added to the cells, incubated for 4 h, and the reaction was terminated by removal of the supernatant and addition of 100 μl DMSO to dissolve the formazan product. After 0.5 h, the optical density (OD) of each well was measured at 490 nm using a plate reader (ELx808, BioTek Instruments, City, ST, U.S.A.).

### BrdU cell proliferation assay

By measuring 5-Bromo-2-deoxyuridine (BrdU) incorporation, DNA synthesis in proliferating cells was determined. BrdU assays were conducted at 24 and 48 h after NHAs were transfected with the indicated vectors or treated with TrkB-IgG (1 μg/ml). Cells were seeded in 96-well culture plates at a density of 2 × 10^3^ cells/well, cultured for 48 h, then incubated with a final concentration of 10 μM BrdU (BD Pharmingen, San Diego, CA, U.S.A.) for 2 h. When the incubation period ended, the medium was removed, the cells were fixed for 30 min at RT, incubated with peroxidase-coupled anti-BrdU antibody (Sigma–Aldrich) for 60 min at RT, washed three times with PBS, incubated with peroxidase substrate (tetramethylbenzidine) for 30 min, and the 450-nm absorbance values were measured for each well. Background BrdU immunofluorescence was determined in cells not exposed to BrdU but stained with the BrdU antibody.

### Colony formation assay

NHAs were seeded and transfected with BDNF vector or si-BDNF or *miR-211* mimics or *miR-211* inhibitor or treated with TrkB-IgG (1 μg/ml). Cells were suspended in astrocyte medium containing 0.35% low-melting agarose and plated on to 0.6% agarose in six-well culture plates at a density of 1 × 10^5^ cells per dish. The plates were incubated for 2 weeks at 37°C in a 5% CO_2_ incubator, and the number of colonies was counted after staining with 0.1% Crystal Violet solution. Colonies with more than 50 cells were manually counted.

### Luciferase reporter assay

HEK293 cells were seeded on to a 24-well plate. A wild-type and mutated BDNF 3′-UTR (wt-BDNF and mut-BDNF containing a 6-bp mutation in the predicted binding sites of *miR-211*) luciferase reporter gene vector was constructed. After culturing overnight, cells were co-transfected with the indicated vectors, and *miR-211* mimics and *miR-211* inhibitor, respectively. Luciferase assays were performed 48 h after transfection using the Dual Luciferase Reporter Assay System (Promega, WI, U.S.A.).

### ELISA assays

The concentrations of BNDF, IL-6, and IL-1β in the astrocyte culture medium were measured using ELISA kits (R&D Systems, U.S.A.) according to the manufacturer’s instructions. Each experiment was repeated three times.

### Statistics analysis

Data from three independent experiments were presented as mean ± S.D., processed using SPSS 17.0 statistical software (SPSS, U.S.A.). Variance (ANOVA) followed by Tukey’s multiple comparison test or independent sample *t* test were used for statistically analyzed. Paired Student’s *t* test was used to compare the expression of *miR-211* and BDNF. *P*-values of <0.05 were considered statistically significant. Each experiment included at least three replicates per condition.

## Results

### LPS stimulation activated release of inflammation-related factors and BDNF expression in NHAs

**Figure 1:**
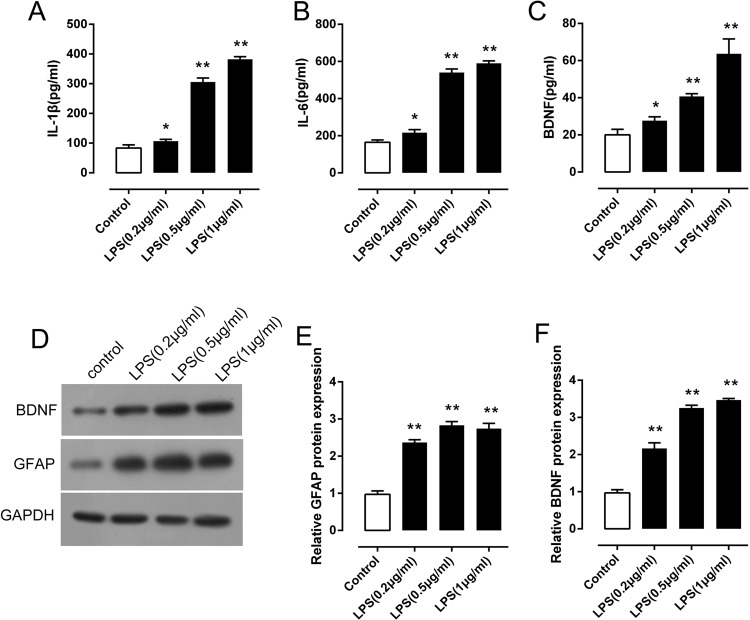
LPS stimulation activated release of inflammation-related factors and BDNF expression in NHAs (**A**–**C**) NHAs were treated with a series of doses of LPS (0.2, 0.5, and 1 μg/ml); the contents of BNDF, IL-6, and IL-1β in the astrocyte culture medium were measured using ELISA kits. (**D–F**) The protein levels of BDNF and GFAP in the astrocyte culture medium were measured using Western blot assays. The data are presented as mean ± S.D. of three independent experiments; *P<0.05, **P<0.01.

To test the potential function of BDNF in the regulation of NHAs, we established astroglial cultures and examined the effect of BDNF on LPS-induced *in vitro* injury model [23–25]. NHAs were exposed to a series of doses of LPS (0.2, 0.5, and 1 μg/ml); the contents of inflammation-related factors, IL-6 and IL-1β, and BDNF in culture medium were determined using ELISA. LPS induces rapid release of proinflammatory cytokines of immune cells and astrocytes [27]. Consistently, we observed an up-regulated content of IL-6 and IL-1β in a dose-dependent manner in culture medium (Figure 1A,B). In addition, BDNF content in culture medium was also increased (Figure 1C). Glial fibrillary acidic protein (GFAP) is considered a marker of astrocytes as well as of neuronal damage [28]. To verify the establishment of LPS-induced *in vitro* injury model, we further monitored the protein levels of GFAP and BDNF in LPS-stimulated NHAs. Upon LPS stimulation, the protein levels of GFAP and BDNF were continually increased in a dose-dependent manner (Figure 1D–F). These data suggested that LPS-induced *in vitro* injury model was successfully established.

### Effects of BDNF on NHA proliferation

**Figure 2:**
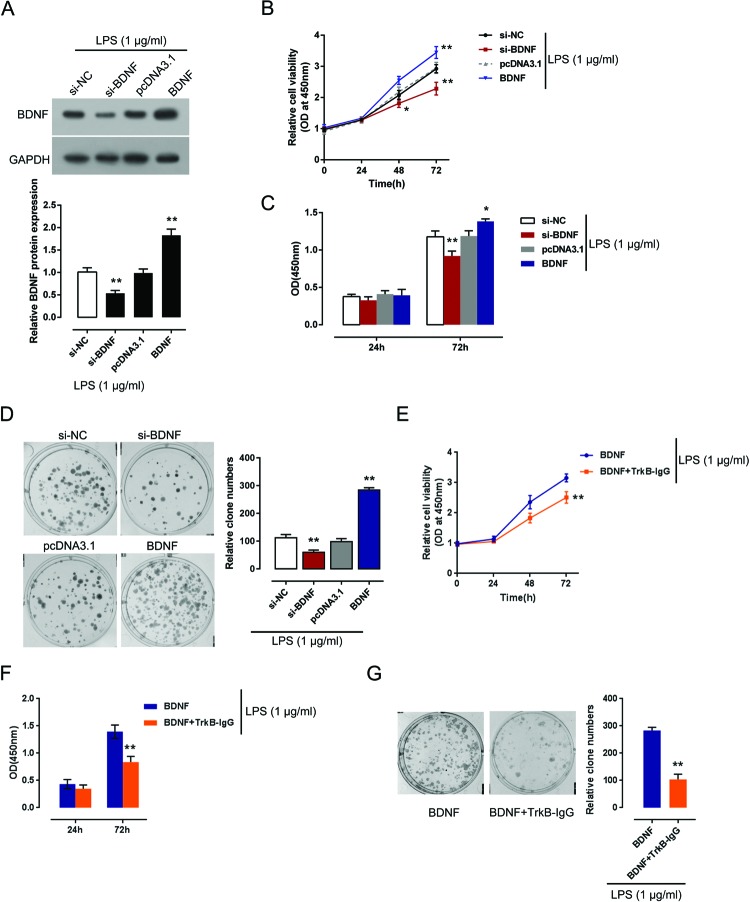
Effects of BDNF on NHA proliferation (**A**) Si-BDNF or BDNF vector was transfected into LPS-treated NHAs to achieve BDNF overexpression or inhibition, as verified using Western blot assays. (**B,C**) The proliferation of NHA was determined using MTT and BrdU assays. (**D**) The colony formation capability of NHA was determined using colony formation assays. (**E**–**G**) BDNF overexpressed NHAs were treated with LPS in absence or presence of TrkB-IgG (1 μg/ml). The MTT (E) assay, BrdU assay (F), and colony formation assay (G) were performed. The data are presented as mean ± S.D. of three independent experiments; *P<0.05, **P<0.01.

To evaluate the functions of BDNF in the regulation of NHA proliferation, we achieved BDNF knockdown or overexpression by transfection of si-BDNF or BDNF vector, as verified using Western blot assays (Figure 2A). The cell viability and DNA synthesis capability of si-BDNF or BDNF vector transfected NHA was determined using MTT and BrdU assays upon 1 μg/ml LPS stimulation. Results showed that BDNF knockdown significantly suppressed NHA proliferation, whereas BDNF overexpression promoted NHA proliferation (Figure 2B,C). Colony formation assays also revealed that BDNF overexpression promoted the colony formation capability of NHA, whereas BDNF knockdown exerted the opposite effect (Figure 2D). To further investigate whether NHA proliferation is associated with BDNF release, we utilized TrkB-IgG to scavenge BDNF and then detected NHA proliferation in response to LPS stimulation. As shown in Figure 2E–G, TrkB-IgG effectively inhibited BDNF overexpression promoted proliferation and colony formation capability of NHA. These data indicated that BDNF positively regulates NHA proliferation upon LPS stimulation.

### *MiR-211* inhibited BDNF expression through direct targetting

**Figure 3:**
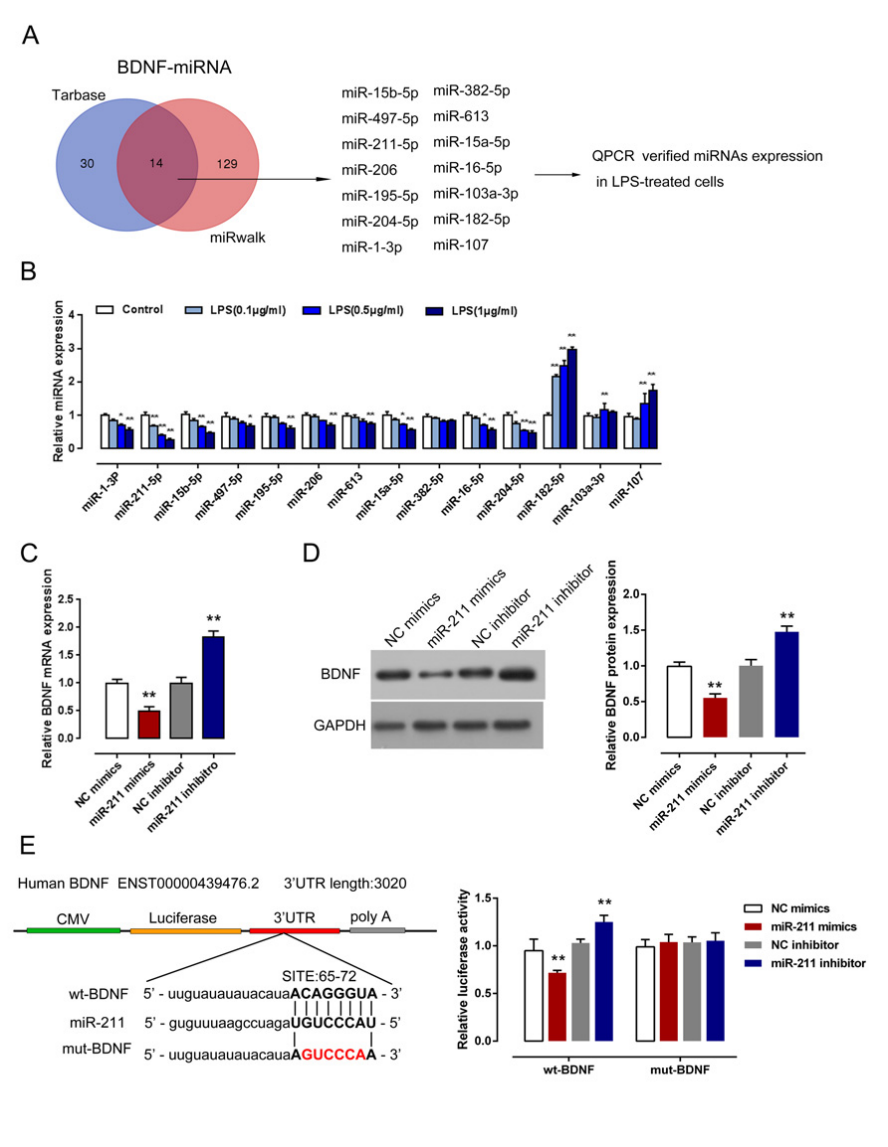
*MiR-211* inhibited BDNF expression through direct targetting (**A**) Online tools including Tarbase and miRWalk were employed to screen for the candidate miRNAs of BDNF. (**B**) The expression levels of the candidate miRNAs in LPS-stimulated NHAs were determined using real-time PCR assays. (**C,D**) NHAs were transfected with *miR-211* mimics or *miR-211* inhibitor; the mRNA and protein levels of BDNF in transfected NHAs were determined using qRT-PCR (C) and Western blot assays (D). (**E**) A wild-type and mutated BDNF 3′-UTR (wt-BDNF and mut-BDNF containing a 6-bp mutation in the predicted binding sites of *miR-211*) luciferase reporter gene vector was constructed. After culturing overnight, cells were cotransfected with the indicated vectors and *miR-211* mimics and *miR-211* inhibitor, respectively. Luciferase activity was determined using the dual luciferase reporter assay system. The data are presented as mean ± S.D. of three independent experiments; *P<0.05; **P<0.01.

According to previous studies, miRNAs affect astrogliosis and astrocyte proliferation through targetting diverse downstream genes [22,26]. To investigate the mechanism by which BDNF affects NHA proliferation, we used online tools including Tarbase and miRWalk to search for the candidate miRNAs which might regulate BDNF expression. After cross-contrast with the results of two software predictions, 14 candidate miRNAs were chosen for further verification (*miR-15b, miR-497, miR-211, miR-206, miR-195, miR-204, miR-1s, miR-382, miR-613, miR-15a, miR-16, miR-103a, miR-182*, and *miR-107*, Figure 3A). Upon LPS stimulation, the expression of the indicated candidate miRNAs was monitored; amongst all the candidate miRNAs, *miR-211* expression was inhibited by LPS stimulation in a dose-dependent manner (Figure 3B). Next, we verified *miR-211* regulation of BDNF using qRT-PCR and Western blot assays. In *miR-211* mimics transfected NHAs, *BDNF* mRNA and protein levels were significantly reduced, whereas *miR-211* inhibitor transfection increased *BDNF* mRNA protein levels (Figure 3C,D). To verify the association between *miR-211* and BDNF, luciferase assays were further employed. A wild-type and mutated BDNF 3′-UTR (wt-BDNF and mut-BDNF containing a 6-bp mutation in the predicted binding sites of *miR-211*) luciferase reporter gene vector was constructed (Figure 3E). The indicated vectors were cotransfected with *miR-211* mimics or *miR-211* inhibitor into HEK293 cells; the luciferase activity was then determined. Results showed that *miR-211* mimics significantly suppressed the luciferase activity in wt-BDNF vector, whereas *miR-211* inhibitor amplified the luciferase activity; after mutation in the predicted binding site of *miR-211*, changes of the luciferase activity were abolished (Figure 3E). These data suggested that *miR-211* could inhibit BDNF expression, partially through binding to the 3′-UTR in BDNF.

### Effects of *miR-211* on NHA proliferation

**Figure 4:**
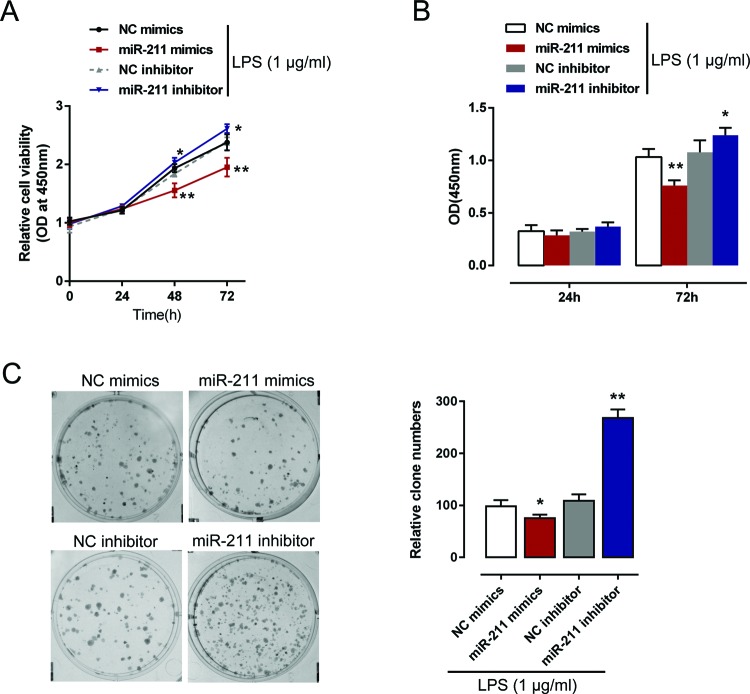
Effects of *miR-211* on NHA proliferation LPS-treated NHAs were transfected with *miR-211* mimics or *miR-211* inhibitor. (**A,B**) The cell proliferation was determined using MTT and BrdU assays; (**C**) the colony formation capability of NHA was determined using colony formation assays. The data are presented as mean ± S.D. of three independent experiments; *P<0.05, **P<0.01.

After confirming the association between *miR-211* and BDNF, we further evaluated the effect of *miR-211* on NHA proliferation. MTT, BrdU, colony formation, and Transwell assays were employed on *miR-211* mimics or *miR-211* inhibitor transfected NHAs upon 1 μg/ml LPS stimulation. Contrary to BDNF, ectopic *miR-211* expression significantly suppressed NHA proliferation, whereas *miR-211* inhibition promoted NHA proliferation (Figure 4A–C). These data suggested that *miR-211* might bind to the 3′-UTR of BDNF to inhibit its expression, thus to suppress NHA proliferation upon LPS stimulation.

### PI3K/Akt pathway was involved in *miR-211* regulation of BDNF expression

**Figure 5:**
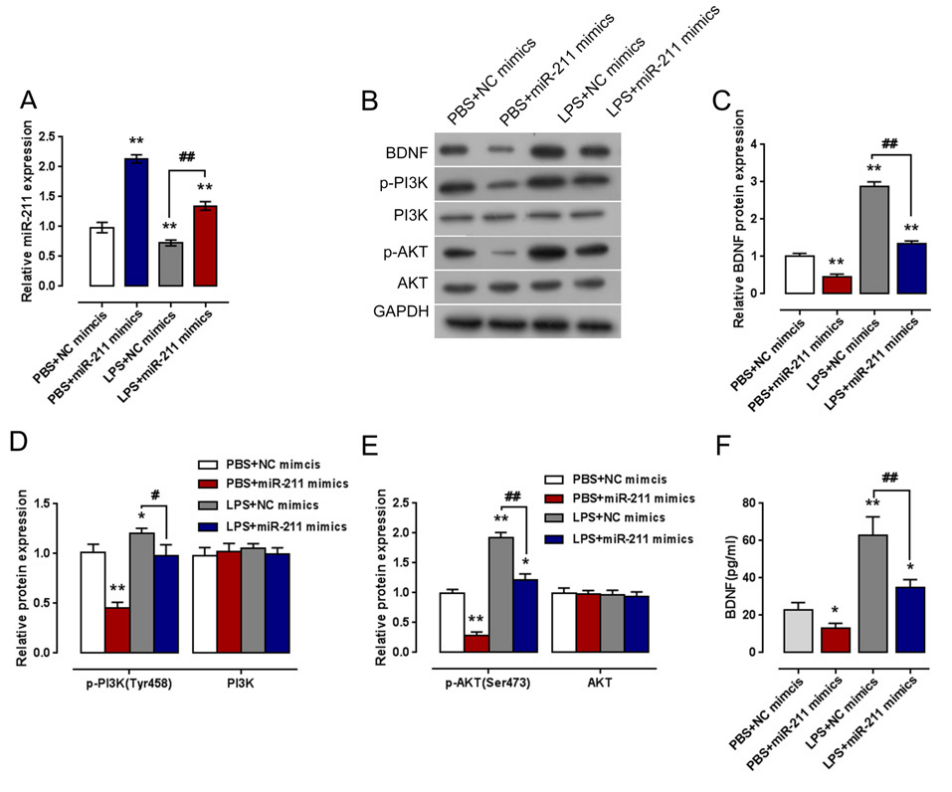
PI3K/Akt pathway was involved in *miR-211* mimics regulation of BDNF expression NHAs were transfected with *miR-211* mimics with the presence or absence of LPS stimulation. (**A**) The *miR-211* expression was determined by qRT-PCR. (**B**–**E**) The protein levels of BDNF, PI3K, *p*-PI3K (Tyr^458^), Akt, and *p*-Akt (Ser^473^) in NHAs were detected by performing immunoblotting. (**F**) The content of BDNF in NHAs was detected using ELISA assays. The data are presented as mean ± S.D. of three independent experiments; *P<0.05, **P<0.01 compared with PBS + NC mimics group; ^#^*P*<0.05, ^##^*P*<0.01 compared with LPS + NC mimics.

As we mentioned, BDNF exerts neuroprotective and growth-promoting effects on a variety of neuronal populations after injury through PI3K/Akt pathway. Here, we also investigated whether PI3K/Akt pathway is involved in *miR-211*/BDNF regulation of NHA proliferation. NHAs were transfected with *miR-211* mimics or *miR-211* inhibitors in the presence or absence of LPS stimulation; the *miR-211* level and protein levels of BDNF, PI3K, *p*-PI3K, Akt, and *p*-Akt were determined using qRT-PCR and Western blot assays, respectively. Results showed that *miR-211* expression was significantly increased by *miR-211* mimics and reduced by LPS stimulation; the suppressive effect of LPS on *miR-211* expression could be reversed by ectopic *miR-211* expression (Figure 5A). Moreover, the protein levels of BDNF, p-PI3K, and p-Akt were significantly reduced by ectopic *miR-211* expression without obvious changes in total PI3K and Akt proteins, whereas increased by LPS stimulation; the suppressive effect of *miR-211* on the indicated proteins could be partially reversed by LPS stimulation (Figure 5B–E). Further, ELISA assays were used to determine BDNF content in NHAs culture medium under the same circumstances. Results showed that *miR-211* reduced BDNF release, LPS induced BDNF release; the suppressive effect of *miR-211* on BDNF release could be partially reversed by LPS stimulation (Figure 5F).

**Figure 6:**
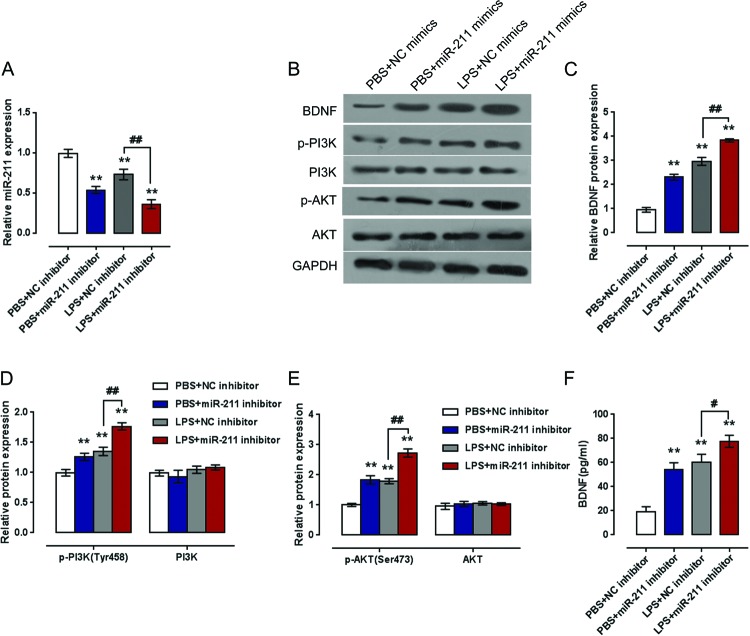
PI3K/Akt pathway was involved in *miR-211* inhibitor regulation of BDNF expression NHAs were transfected with *miR-211* inhibitor with the presence or absence of LPS stimulation. (**A**) *MiR-211* expression was determined by qRT-PCR. (**B**–**E**) The protein levels of BDNF, PI3K, *p*-PI3K (Tyr^458^), Akt, and *p*-Akt (Ser^473^) in NHAs were detected by immunoblotting. (**F**) The content of BDNF in NHAs culture medium was detected using ELISA assays. The data are presented as mean ± S.D. of three independent experiments; ffsflp[sedk **P<0.01, V.S. PBS + NC mimics group; ^#^*P*<0.05, ^##^*P*<0.01, V.S. LPS + NC mimics.

In contrast, *miR-211* expression was significantly decreased by *miR-211* inhibitors and further reduced by LPS stimulation (Figure 6A). After suppressing *miR-211* expression, the protein levels of BDNF, p-PI3K, and p-Akt were increased without obvious changes in total PI3K and Akt proteins. LPS stimulation further up-regulated the indicated proteins, (Figure 6B–E). LPS stimulation also further enhanced the promoting effect of *miR-211* inhibitor on the release of BDNF (Figure 6F).

## Discussion

In the present study, we established an LPS-induced *in vitro* injury model of astroglial culture to investigate the roles and molecular mechanism of BDNF in the regulation of NHA proliferation. In response to LPS stimulation, the contents of IL-1β, IL-6, and BDNF were promoted; BDNF promoted the proliferation of NHA upon LPS stimulation. Through binding to the 3′-UTR of BDNF, *miR-211* inhibited BDNF expression and further suppressed NHA proliferation. Further, we revealed the involvement of PI3K/Akt pathway in the regulation of NHA proliferation.

Astrocytes are the most abundant glial cells in the CNS, which are essential for various structural and physiological functions [29,30]. After SCI, local environment undergoes profound biochemical and cellular changes which start with an immediate influx of inflammatory cells into the injured spinal cord that releases a host of cytokines and chemokines causing exitotoxicity and cell damage. In the meantime, SCI triggers astrocytes to become reactive and initiate astrogliosis [31,32]. Reactive astrogliosis is characterized by the proliferation and hypertrophy of astrocytes, which eventually leads to scar formation via the effects of neurotrophic factors such as BDNF [33], or activation of signaling pathways such as STAT3, transforming growth factors-β (TGF-β/Smad) and PI3K [12,32,34–37]. Upon injury, astrocytes undergo phenotypic and morphologic changes. They increase their expression of intermediate filaments such as GFAPs, nestin, and vimentin [29,38]. Reactive astrocytes also contribute to the release of pro- and anti-inflammatory cytokines such as TGF-β, tumor necrosis factor-α (TNF-α), and interleukins (IL-1 and IL-6) that modulate inflammation and secondary injury mechanisms. In the present study, we have evaluated the release of BDNF, IL-1β, and IL-6 in LPS-stimulated NHAs. Consistent with previous studies, LPS stimulation significantly increased the contents of BDNF, IL-1β, and IL-6 in NHAs. In addition, the protein levels of GFAP and BDNF in LPS-simulated NHAs were also increased, which further indicated the successful establishment of LPS-induced *in vitro* injury model of astroglial cultures.

During development, growth-permissive neurotrophic factors, including BDNF, allow axons to lengthen and extend toward appropriate targets in the correct numbers. In the adult, these factors contribute to neuronal survival, axonal plasticity, and synaptic function, including neurotransmitter availability [39,40]. BDNF can enhance regeneration and sprouting of injured axons in the spinal cord [41] or increased remyelination of injured axons. However, there is some conflicting evidence associated with BDNF-based treatment that BDNF might contribute to hyperproliferation of NHA, which leads to the glial scar formation after SCI. Co-operating with its receptor TrkB, BDNF exerts neuroprotective and growth-promoting effects on a variety of neuronal populations after injury. It has been well documented that BDNF/TrkB pathway is involved in astrocytes activation [42,43]. Moreover, Aroeira et al. [44] found that the effect of BDNF on astrocytes was dependent on TrkB-T1 rather than TrkB full length. In the present study, we have evaluated the functions of BDNF in the regulation of NHA proliferation. Consistent with the previous studies, we observed that BDNF dramatically promoted NHA proliferation and TrkB-IgG effectively blocked the effect of BDNF on NHA proliferation. Inhibiting BDNF expression in NHA after SCI presents an efficient strategy for suppressing glial scar formation.

In the pathophysiology of SCI, the secondary biological processes involving changes in gene expression become more and more important. Within these changes, miRNA expression participates in some of the pathophysiological processes in SCI [45]. Several studies have described the transient expression of miRNAs in SCI, some of them related to inflammation and apoptosis and others to functional recovery and regeneration. Overexpression of *miR-145* reduced the size of astrocytes and the number of related cellular processes, as well as cell proliferation and migration [46]. Another study demonstrated that astrocytes adjacent to the lesion area expressed high levels of *miR-21* whereas astrocytes in uninjured spinal cord expressed low levels of *miR-21*; overexpression of *miR-21* in astrocytes attenuated the hypertrophic response to SCI [47]. MiRNAs bind to the 3′-UTR of target mRNAs via imperfect base pairing followed by either inhibition of translation or target degradation [20,21]. According to previous studies, BDNF was associated with astrocyte viability or differentiation [48,49]. To search for the candidate miRNAs which might inhibit BDNF expression in NHA upon LPS stimulation, we employed online tools, including Tarbase and miRWalk. Amongst the candidate miRNAs, *miR-211* expression was the most strongly suppressed by LPS stimulation. Further, we confirmed the association between *miR-211* and BDNF; through direct binding to the 3′-UTR of BDNF, *miR-211* significantly down-regulated *BDNF* mRNA and protein expression in NHAs. Moreover, *miR-211* exerted the opposite functions to BDNF in the regulation of NHA proliferation; ectopic *miR-211* expression significantly suppressed NHA proliferation, while inhibition of *miR-211* expression promoted NHA proliferation.

Several signaling pathways can affect the neuroprotective outcomes, including PI3K/Akt signaling [13,17]. In the present study, we also investigated whether PI3K/Akt signaling can affect LPS-induced BDNF expression. The protein levels of BDNF, p-PI3K, and p-Akt could be significantly reduced by *miR-211*, whereas increased by LPS stimulation, without obvious changes in total PI3K and Akt proteins. Moreover, the suppressive effect of *miR-211* on the indicated proteins could be partially reversed by LPS stimulation. Contrary to the epic *miR-211* expression, inhibiting *miR-211* expression showed an opposite effect on cell proliferation and indicated protein expression in response to LPS stimulation. These data indicated that PI3K/Akt pathway was involved in *miR-211* regulation of LPS-induced BDNF expression.

Taken together, *miR-211*/BDNF axis regulates LPS-induced NHA proliferation through PI3K/AKT pathway; *miR-211*/BDNF might serve as a promising target in the strategy against reactive astrocyte proliferation after SCI.

## Abbreviations

BDNF: brain-derived neurotrophic factor
BrdU: 5-Bromo-2-deoxyuridine
CNS: central nervous system
GFAP: glial fibrillary acidic protein
IL: interleukin
NHA: normal human astrocyte
SCI: spinal cord injury
TGF-β: transforming growth factor-β
TrkB: tropomyosin-related kinase B
